# Supplementary material for: A Likelihood Approach for Real-Time Calibration of Stochastic Compartmental Epidemic Models
Source: PLoS Comput Biol. 2017 Jan 17;13(1):e1005257. doi: 10.1371/journal.pcbi.1005257 (PMC5240920; doi:10.1371/journal.pcbi.1005257)
Supplement: S1 File — (TAR.GZ) [file pcbi.1005257.s014.tar.gz › HSPH_Online-SI-Revision/IPoi09/n100-mild/n100-mild_BENCH2_table4.pdf]

1	newly 3 weeks spec	734.	1928.67	{660., 3789.}	167.322	0.355277	newly 3 weeks cumul	3465.	8316.02	{3179., 15625.}	143.879	0.330284	newly 5 weeks spec	287.	930.738	{304., 1830.}	226.058	0.415434	newly 5 weeks cumul	4194.	10588.7	{3919., 20247.}	155.886	0.344018	newly 48 weeks cumul	4721.	12495.4	{4578., 23887.}	167.471	0.356796
2	newly 3 weeks spec	1022.	2395.76	{918., 4102.}	138.329	0.301151	newly 3 weeks cumul	4122.	9631.08	{3895., 16321.}	136.239	0.311694	newly 5 weeks spec	367.	1281.63	{433., 2083.}	249.26	0.427311	newly 5 weeks cumul	5105.	12669.5	{4942., 21355.}	150.314	0.326805	newly 47 weeks cumul	5925.	15825.4	{6013., 26478.}	168.556	0.346541
3	newly 3 weeks spec	703.	1098.63	{511., 1782.}	69.9407	0.212701	newly 3 weeks cumul	2922.	5441.8	{2606., 8506.}	92.1587	0.25242	newly 5 weeks spec	387.	447.366	{198., 713.}	44.7407	0.175891	newly 5 weeks cumul	3733.	6584.73	{3139., 10436.}	83.7311	0.235729	newly 50 weeks cumul	4191.	7341.78	{3402., 11718.}	82.9711	0.234353
4	newly 3 weeks spec	301.	546.964	{206., 1131.}	98.1214	0.251614	newly 3 weeks cumul	2102.	3414.6	{1310., 7028.}	81.7187	0.227186	newly 5 weeks spec	86.	162.873	{52., 351.}	105.074	0.264348	newly 5 weeks cumul	2358.	3875.35	{1454., 8014.}	83.3326	0.229686	newly 54 weeks cumul	2427.	4053.95	{1496., 8407.}	85.3759	0.232246
5	newly 3 weeks spec	828.	1098.63	{511., 1782.}	54.1534	0.187929	newly 3 weeks cumul	3560.	5441.8	{2606., 8506.}	65.9414	0.205501	newly 5 weeks spec	386.	447.366	{198., 713.}	44.8755	0.175973	newly 5 weeks cumul	4530.	6584.73	{3139., 10436.}	60.9767	0.197202	newly 50 weeks cumul	5288.	7341.78	{3402., 11718.}	56.6617	0.189883
6	newly 3 weeks spec	764.	1547.38	{616., 3105.}	111.066	0.266445	newly 3 weeks cumul	3259.	6942.6	{2959., 13400.}	117.455	0.278778	newly 5 weeks spec	315.	713.458	{264., 1438.}	132.526	0.291244	newly 5 weeks cumul	4097.	8710.5	{3621., 16934.}	117.676	0.277576	newly 48 weeks cumul	4608.	10133.3	{4078., 19709.}	124.514	0.287016
7	newly 3 weeks spec	276.	1256.44	{548., 2353.}	355.233	0.583332	newly 3 weeks cumul	1666.	6693.94	{3053., 12924.}	301.797	0.536237	newly 5 weeks spec	53.	439.192	{196., 863.}	728.665	0.842587	newly 5 weeks cumul	1818.	7879.13	{3600., 15130.}	333.396	0.568075	newly 53 weeks cumul	1893.	8494.96	{3831., 16390.}	348.757	0.582388
8	newly 3 weeks spec	397.	1558.96	{598., 2725.}	293.244	0.48801	newly 3 weeks cumul	2114.	6519.96	{2812., 11853.}	209.37	0.412259	newly 5 weeks spec	163.	793.533	{275., 1335.}	386.83	0.54169	newly 5 weeks cumul	2526.	8428.82	{3522., 15053.}	234.397	0.43442	newly 47 weeks cumul	2724.	10185.4	{4107., 17856.}	274.32	0.470776
9	newly 3 weeks spec	565.	1618.51	{573., 3244.}	190.368	0.338022	newly 3 weeks cumul	2870.	7761.71	{3091., 15977.}	173.756	0.341211	newly 5 weeks spec	200.	653.491	{221., 1280.}	229.873	0.368625	newly 5 weeks cumul	3444.	9454.	{3655., 19392.}	177.935	0.340851	newly 52 weeks cumul	3765.	10487.9	{3991., 21412.}	182.092	0.343013
10	newly 3 weeks spec	792.	1528.45	{534., 3259.}	106.977	0.258949	newly 3 weeks cumul	3481.	6506.32	{2531., 13225.}	100.197	0.252274	newly 5 weeks spec	358.	791.872	{265., 1711.}	132.426	0.295112	newly 5 weeks cumul	4318.	8394.26	{3216., 17191.}	106.813	0.26059	newly 46 weeks cumul	4964.	10206.5	{3870., 21123.}	116.582	0.272711
11	newly 3 weeks spec	702.	2686.31	{1091., 4780.}	282.976	0.508133	newly 3 weeks cumul	3890.	11064.7	{5124., 20008.}	185.026	0.408016	newly 5 weeks spec	225.	1502.87	{498., 2323.}	567.943	0.682376	newly 5 weeks cumul	4534.	14564.9	{6415., 25772.}	221.679	0.446682	newly 50 weeks cumul	4756.	18051.9	{7392., 30689.}	279.706	0.498101
12	newly 3 weeks spec	1213.	2688.85	{1022., 5087.}	127.08	0.293303	newly 3 weeks cumul	4897.	10669.2	{4549., 19692.}	121.675	0.292786	newly 5 weeks spec	613.	1509.2	{594., 2966.}	149.921	0.317066	newly 5 weeks cumul	6341.	14209.1	{5907., 26571.}	127.849	0.298996	newly 46 weeks cumul	8505.	18182.5	{7393., 34676.}	119.195	0.280929
13	newly 3 weeks spec	527.	1171.1	{507., 1975.}	126.949	0.303775	newly 3 weeks cumul	2892.	5910.42	{2735., 9728.}	109.737	0.277285	newly 5 weeks spec	244.	452.2	{194., 759.}	95.2295	0.251842	newly 5 weeks cumul	3457.	7084.24	{3235., 11674.}	110.411	0.277925	newly 51 weeks cumul	3655.	7814.86	{3552., 12958.}	118.548	0.291445
14	newly 3 weeks spec	361.	781.411	{207., 1416.}	134.285	0.274918	newly 3 weeks cumul	1836.	4339.8	{1140., 7872.}	148.437	0.299753	newly 5 weeks spec	115.	256.857	{69., 448.}	141.335	0.273986	newly 5 weeks cumul	2169.	5042.98	{1339., 9080.}	145.283	0.293115	newly 53 weeks cumul	2408.	5364.66	{1421., 9645.}	136.854	0.280991
15	newly 3 weeks spec	612.	3238.98	{1199., 6821.}	429.246	0.628374	newly 3 weeks cumul	3189.	12955.2	{5632., 26628.}	306.246	0.543551	newly 5 weeks spec	228.	1720.02	{571., 3545.}	654.393	0.74273	newly 5 weeks cumul	3718.	17062.8	{7059., 35264.}	358.925	0.584486	newly 50 weeks cumul	4085.	20993.	{8260., 43538.}	413.904	0.621641
16	newly 3 weeks spec	1144.	1304.55	{493., 2318.}	55.4589	0.214326	newly 3 weeks cumul	4245.	5704.13	{2314., 10244.}	58.7438	0.190867	newly 5 weeks spec	573.	623.85	{237., 1135.}	56.3962	0.226635	newly 5 weeks cumul	5611.	7244.8	{2897., 13086.}	57.9184	0.195694	newly 46 weeks cumul	6762.	8592.68	{3386., 15621.}	58.245	0.199813
17	newly 3 weeks spec	602.	1609.68	{673., 2658.}	169.843	0.335352	newly 3 weeks cumul	3587.	7888.67	{3601., 13539.}	123.711	0.276003	newly 5 weeks spec	237.	630.684	{239., 1053.}	168.924	0.321239	newly 5 weeks cumul	4153.	9528.75	{4237., 16301.}	132.882	0.287977	newly 52 weeks cumul	4431.	10526.3	{4599., 17873.}	140.633	0.298088
18	newly 3 weeks spec	80.	366.377	{145., 654.}	357.972	0.580532	newly 3 weeks cumul	978.	2701.83	{1116., 4756.}	176.485	0.373672	newly 5 weeks spec	21.	85.0189	{33., 163.}	305.211	0.530512	newly 5 weeks cumul	1011.	2966.79	{1223., 5223.}	193.451	0.398645	newly 56 weeks cumul	1018.	3048.57	{1242., 5390.}	199.466	0.407413
19	newly 3 weeks spec	761.	2860.19	{1145., 5605.}	276.892	0.49287	newly 3 weeks cumul	3302.	11338.3	{5214., 21514.}	244.209	0.471514	newly 5 weeks spec	360.	1528.45	{534., 3259.}	325.573	0.526316	newly 5 weeks cumul	4108.	14984.4	{6600., 29134.}	265.535	0.490112	newly 47 weeks cumul	4733.	18684.6	{7939., 37032.}	295.461	0.516509
20	newly 3 weeks spec	252.	780.383	{195., 1971.}	219.858	0.366426	newly 3 weeks cumul	2135.	5336.81	{1457., 14115.}	164.027	0.315546	newly 5 weeks spec	37.	186.638	{39., 471.}	407.648	0.517175	newly 5 weeks cumul	2324.	5903.83	{1598., 15542.}	168.069	0.319271	newly 56 weeks cumul	2336.	6081.53	{1630., 16087.}	173.499	0.323951
21	newly 3 weeks spec	232.	366.377	{145., 654.}	77.9278	0.228211	newly 3 weeks cumul	1801.	2701.83	{1116., 4756.}	68.0314	0.20482	newly 5 weeks spec	64.	85.0189	{33., 163.}	61.7925	0.213947	newly 5 weeks cumul	1977.	2966.79	{1223., 5223.}	68.2481	0.205734	newly 56 weeks cumul	2067.	3048.57	{1242., 5390.}	66.4522	0.202982
22	newly 3 weeks spec	1379.	2340.06	{1081., 4501.}	80.6842	0.221024	newly 3 weeks cumul	5215.	9547.75	{4570., 17830.}	88.4826	0.234659	newly 5 weeks spec	709.	1244.5	{504., 2579.}	88.0276	0.233367	newly 5 weeks cumul	6926.	12524.5	{5875., 23745.}	87.4545	0.231731	newly 46 weeks cumul	9186.	15660.7	{7227., 30179.}	80.7255	0.221283
23	newly 3 weeks spec	467.	2482.16	{903., 5063.}	431.513	0.620185	newly 3 weeks cumul	3051.	10848.9	{4480., 21828.}	255.898	0.470503	newly 5 weeks spec	143.	1134.51	{386., 2330.}	693.363	0.77038	newly 5 weeks cumul	3445.	13671.2	{5432., 27617.}	296.841	0.509058	newly 51 weeks cumul	3590.	15859.3	{6208., 32082.}	341.764	0.549751
24	newly 3 weeks spec	99.	350.14	{118., 689.}	254.566	0.446651	newly 3 weeks cumul	1045.	2454.9	{864., 4811.}	143.045	0.312604	newly 5 weeks spec	29.	84.02	{27., 180.}	193.448	0.363726	newly 5 weeks cumul	1106.	2711.12	{942., 5315.}	151.761	0.322369	newly 55 weeks cumul	1133.	2790.98	{966., 5499.}	152.966	0.323843
25	newly 3 weeks spec	1111.	3981.21	{1988., 6600.}	258.345	0.512186	newly 3 weeks cumul	4640.	14691.1	{7229., 22386.}	216.618	0.468742	newly 5 weeks spec	633.	2538.68	{1101., 4535.}	301.056	0.537193	newly 5 weeks cumul	6102.	20429.2	{10629., 32635.}	234.796	0.489046	newly 46 weeks cumul	7554.	28934.2	{13700., 48005.}	283.032	0.531894
26	newly 3 weeks spec	720.	2460.3	{1149., 4379.}	241.708	0.453322	newly 3 weeks cumul	3694.	10212.1	{5217., 17800.}	176.889	0.379963	newly 5 weeks spec	332.	1270.74	{504., 2165.}	282.753	0.489484	newly 5 weeks cumul	4491.	13254.6	{6541., 23134.}	195.387	0.402018	newly 48 weeks cumul	5251.	16150.8	{7733., 28115.}	207.801	0.414303
27	newly 3 weeks spec	1036.	3694.52	{1613., 6540.}	256.614	0.498601	newly 3 weeks cumul	4214.	13725.8	{6586., 23225.}	225.719	0.4721	newly 5 weeks spec	467.	2305.91	{850., 5016.}	393.771	0.615078	newly 5 weeks cumul	5302.	18948.9	{8711., 32616.}	257.391	0.507026	newly 46 weeks cumul	6355.	26369.2	{11168., 51965.}	314.936	0.556104
28	newly 3 weeks spec	1038.	1986.14	{773., 4159.}	105.901	0.252342	newly 3 weeks cumul	4164.	8202.23	{3465., 16753.}	105.929	0.25717	newly 5 weeks spec	683.	1008.44	{339., 2197.}	84.2589	0.251387	newly 5 weeks cumul	5692.	10645.5	{4319., 22013.}	100.095	0.248379	newly 47 weeks cumul	8123.	12860.5	{5046., 26600.}	82.0243	0.229402
29	newly 3 weeks spec	584.	1130.98	{427., 2207.}	105.797	0.23354	newly 3 weeks cumul	2514.	5291.88	{2233., 10435.}	117.497	0.261029	newly 5 weeks spec	328.	477.829	{162., 924.}	83.0532	0.237898	newly 5 weeks cumul	3268.	6512.66	{2664., 12857.}	108.616	0.244328	newly 49 weeks cumul	4524.	7338.68	{2900., 14321.}	81.7375	0.207855
30	newly 3 weeks spec	466.	2306.6	{1011., 3273.}	394.978	0.594716	newly 3 weeks cumul	2784.	10000.3	{4821., 15141.}	259.205	0.480488	newly 5 weeks spec	120.	1078.66	{461., 1474.}	798.884	0.831639	newly 5 weeks cumul	3116.	12671.3	{6003., 18747.}	306.652	0.526301	newly 51 weeks cumul	3221.	14802.6	{6888., 21543.}	359.566	0.573167
31	newly 3 weeks spec	874.	2180.43	{956., 3813.}	151.999	0.341809	newly 3 weeks cumul	3903.	9059.3	{4387., 15387.}	134.368	0.328144	newly 5 weeks spec	482.	1109.89	{404., 1894.}	135.91	0.293327	newly 5 weeks cumul	4981.	11734.1	{5440., 19821.}	138.106	0.327761	newly 48 weeks cumul	5893.	14229.6	{6409., 23789.}	144.104	0.329749
32	newly 3 weeks spec	1191.	2117.68	{852., 4361.}	94.0315	0.243273	newly 3 weeks cumul	4640.	8718.36	{3699., 17247.}	98.5423	0.25154	newly 5 weeks spec	844.	1096.06	{393., 2255.}	72.9026	0.257535	newly 5 weeks cumul	6529.	11342.9	{4626., 22761.}	88.6545	0.236313	newly 46 weeks cumul	9247.	13947.	{5572., 28404.}	75.8507	0.227687
33	newly 3 weeks spec	62.	168.311	{65., 341.}	175.412	0.345708	newly 3 weeks cumul	424.	1452.29	{528., 2769.}	244.638	0.433651	newly 5 weeks spec	6.	34.5111	{8., 88.}	475.185	0.610322	newly 5 weeks cumul	438.	1564.09	{584., 3028.}	258.935	0.449746	newly 56 weeks cumul	438.	1590.16	{604., 3079.}	264.805	0.45613
34	newly 3 weeks spec	175.	1184.39	{395., 2188.}	576.796	0.706868	newly 3 weeks cumul	1326.	6125.	{2131., 11717.}	362.169	0.55597	newly 5 weeks spec	25.	435.982	{128., 820.}	1643.93	1.10255	newly 5 weeks cumul	1434.	7286.21	{2484., 13866.}	408.104	0.59283	newly 52 weeks cumul	1519.	7933.57	{2678., 15003.}	422.289	0.602973
35	newly 3 weeks spec	0.1	189.306	{60., 324.}	189206.	3.14562	newly 3 weeks cumul	47.	1590.43	{537., 2761.}	3283.89	1.39949	newly 5 weeks spec	0.1	35.2653	{8., 71.}	35165.3	2.37287	newly 5 weeks cumul	47.	1707.14	{566., 2979.}	3532.22	1.42973	newly 56 weeks cumul	47.	1735.65	{571., 3058.}	3592.88	1.43706
36	newly 3 weeks spec	307.	305.	{127., 740.}	56.0715	0.24731	newly 3 weeks cumul	1782.	2422.93	{1032., 5627.}	66.0768	0.210863	newly 5 weeks spec	117.	63.6977	{23., 140.}	57.1258	0.407099	newly 5 weeks cumul	2037.	2628.79	{1120., 6082.}	62.4139	0.209425	newly 56 weeks cumul	2462.	2681.14	{1132., 6214.}	54.91	0.219677
37	newly 3 weeks spec	611.	2448.09	{936., 4276.}	300.67	0.520865	newly 3 weeks cumul	3063.	10320.3	{4420., 18030.}	237.065	0.465555	newly 5 weeks spec	219.	1241.62	{448., 2025.}	466.949	0.646084	newly 5 weeks cumul	3644.	13325.5	{5484., 23088.}	265.683	0.492932	newly 49 weeks cumul	4006.	16094.9	{6470., 27802.}	301.77	0.525366
38	newly 3 weeks spec	40.	564.082	{211., 1096.}	1310.2	1.05359	newly 3 weeks cumul	901.	4036.53	{1572., 7747.}	348.006	0.563765	newly 5 weeks spec	6.	129.531	{45., 293.}	2058.84	1.22816	newly 5 weeks cumul	919.	4437.73	{1724., 8621.}	382.887	0.595273	newly 56 weeks cumul	919.	4554.06	{1752., 8906.}	395.545	0.605981
39	newly 3 weeks spec	244.	452.738	{145., 902.}	107.309	0.270012	newly 3 weeks cumul	1717.	2786.69	{913., 5475.}	90.5192	0.25744	newly 5 weeks spec	134.	124.024	{32., 247.}	61.9581	0.313375	newly 5 weeks cumul	1991.	3140.36	{1024., 6220.}	88.2985	0.258589	newly 53 weeks cumul	2147.	3283.12	{1053., 6533.}	85.8219	0.259526
40	newly 3 weeks spec	154.	505.056	{143., 888.}	231.83	0.352264	newly 3 weeks cumul	1047.	3283.22	{1021., 6163.}	215.964	0.345553	newly 5 weeks spec	17.	130.815	{33., 241.}	669.499	0.689877	newly 5 weeks cumul	1131.	3670.19	{1131., 6884.}	226.296	0.354702	newly 55 weeks cumul	1136.	3800.41	{1166., 7185.}	236.092	0.365685
41	newly 3 weeks spec	226.	660.074	{200., 1351.}	195.837	0.348847	newly 3 weeks cumul	1612.	4436.87	{1491., 8748.}	179.272	0.338604	newly 5 weeks spec	99.	161.537	{48., 302.}	92.125	0.237095	newly 5 weeks cumul	1842.	4923.76	{1640., 9801.}	172.34	0.329095	newly 56 weeks cumul	1901.	5080.91	{1682., 10139.}	172.37	0.32873
42	newly 3 weeks spec	285.	949.037	{361., 1705.}	233.242	0.446278	newly 3 weeks cumul	1860.	5211.83	{2061., 8835.}	181.168	0.380049	newly 5 weeks spec	90.	322.352	{121., 562.}	258.416	0.472916	newly 5 weeks cumul	2101.	6081.61	{2373., 10397.}	190.258	0.392131	newly 53 weeks cumul	2198.	6517.94	{2523., 11149.}	197.271	0.401783
43	newly 3 weeks spec	344.	958.222	{284., 1825.}	186.197	0.310554	newly 3 weeks cumul	2328.	6008.33	{2022., 11904.}	164.557	0.305945	newly 5 weeks spec	97.	256.148	{79., 471.}	173.616	0.277043	newly 5 weeks cumul	2595.	6769.54	{2251., 13269.}	167.401	0.306056	newly 56 weeks cumul	2707.	7030.57	{2304., 13812.}	166.655	0.304457
44	newly 3 weeks spec	177.	652.375	{194., 1103.}	270.47	0.423138	newly 3 weeks cumul	1333.	4158.71	{1335., 7076.}	214.883	0.375149	newly 5 weeks spec	67.	176.857	{46., 308.}	176.439	0.323671	newly 5 weeks cumul	1521.	4674.96	{1477., 7966.}	210.846	0.369061	newly 55 weeks cumul	1548.	4859.86	{1517., 8230.}	217.156	0.375986
45	newly 3 weeks spec	708.	1324.04	{455., 2294.}	101.501	0.25613	newly 3 weeks cumul	3626.	6552.17	{2393., 11610.}	94.9336	0.250395	newly 5 weeks spec	308.	516.167	{170., 935.}	88.3387	0.24159	newly 5 weeks cumul	4438.	7900.4	{2826., 13917.}	93.466	0.248354	newly 51 weeks cumul	4856.	8725.19	{3080., 15334.}	95.0521	0.250345
46	newly 3 weeks spec	683.	2855.62	{1077., 5155.}	318.1	0.527563	newly 3 weeks cumul	3807.	12456.4	{5200., 22768.}	227.196	0.448815	newly 5 weeks spec	255.	1309.84	{467., 2311.}	413.661	0.594209	newly 5 weeks cumul	4509.	15713.5	{6318., 28608.}	248.493	0.46787	newly 52 weeks cumul	4663.	18183.	{7125., 32861.}	289.941	0.509843
47	newly 3 weeks spec	364.	1609.68	{673., 2658.}	342.221	0.542052	newly 3 weeks cumul	2257.	7888.67	{3601., 13539.}	249.52	0.45831	newly 5 weeks spec	75.	630.684	{239., 1053.}	740.912	0.806727	newly 5 weeks cumul	2514.	9528.75	{4237., 16301.}	279.028	0.488963	newly 52 weeks cumul	2640.	10526.3	{4599., 17873.}	298.725	0.507945
48	newly 3 weeks spec	674.	3853.84	{1852., 6975.}	471.786	0.705019	newly 3 weeks cumul	3437.	14560.9	{7791., 24880.}	323.651	0.588869	newly 5 weeks spec	214.	2305.33	{1070., 4370.}	977.256	0.962598	newly 5 weeks cumul	4062.	19881.3	{10277., 34804.}	389.445	0.645233	newly 47 weeks cumul	4384.	26509.6	{13109., 47755.}	504.691	0.728928
49	newly 3 weeks spec	230.	631.442	{223., 1029.}	179.211	0.308537	newly 3 weeks cumul	1489.	3690.79	{1343., 5929.}	153.384	0.290632	newly 5 weeks spec	69.	189.674	{65., 322.}	181.159	0.307713	newly 5 weeks cumul	1667.	4229.93	{1518., 6778.}	158.935	0.295567	newly 53 weeks cumul	1729.	4449.21	{1593., 7062.}	162.039	0.297935
50	newly 3 weeks spec	221.	587.814	{161., 1556.}	175.892	0.303116	newly 3 weeks cumul	1420.	3731.37	{1083., 9773.}	172.612	0.308157	newly 5 weeks spec	59.	164.395	{40., 477.}	191.407	0.330145	newly 5 weeks cumul	1550.	4207.47	{1193., 11109.}	180.716	0.317162	newly 54 weeks cumul	1593.	4383.56	{1232., 11662.}	184.295	0.321279
